# Social Factors Associated With Adherence to Preventive Behaviors Related to COVID-19 Among Rural and Semi-urban Communities in Western Maharashtra, India

**DOI:** 10.3389/fpubh.2021.722621

**Published:** 2021-09-08

**Authors:** Suhas P. Shewale, Suvarna Sanjay Sane, Dhammasagar Dnyaneshwar Ujagare, Rais Patel, Sudipto Roy, Sanjay Juvekar, Rewa Kohli, Sampada Bangar, Asha Jadhav, Seema Sahay

**Affiliations:** ^1^Division of Social and Behavioral Research, Indian Council of Medical Research, National AIDS Research Institute, Pune, India; ^2^Krishna Institute of Medical Sciences Deemed To Be University, Karad, India; ^3^Division of Epidemiology and Biostatistics, Indian Council of Medical Research, National AIDS Research Institute, Pune, India; ^4^KEM Hospital Research Centre, Pune, India

**Keywords:** COVID-19, lockdown, handwashing, face-mask, adherence, personal protective measures, rural, India

## Abstract

**Background:** To control the transmission of the coronavirus disease 2019 (COVID-19) infection, the Government of India (GoI) had taken stringent precautionary measures during the lockdown period. This study aimed to explore determinants affecting adherence to protective measures against COVID-19 infection among rural and semi-urban settings of Maharashtra, India.

**Methods:** A cross-sectional telephonic survey among 1,016 adults from randomly selected households was conducted between June 5 and July 16, 2020. The data were explored for knowledge, awareness, practices related to protective measures, and self-risk perception. Socio-demographic and attitudinal correlates of failure to use protective measures against COVID-19 were measured.

**Results:** In the survey, 72% of the participants were men. The mean age was 46 years (SD: 13.8). The main source of information was television (91%); however, information from healthcare providers (65%) and mass media announcements (49%) was trustworthy. Washing hands immediately with soap after returning from outdoors was reported by 95% of the respondents, always using a mask while outdoors by 94%, never attended social gatherings by 91%, always using hand sanitizer while outside by 77%, and 68% of the respondents followed all protective measures. The knowledge score [mean score 20.3 (SD: 2.4) out of 24] was independently associated with the risk of not using protective measures, with each unit increase in knowledge score, the risk of not using protective measures reduced by 16%. No source of income was independently associated with not using protective measures [AOR 1.5 95% *CI* (1.01–2.3)].

**Conclusions:** The COVID-19 public health interventions and behavior change communication strategies should be specifically directed towards the low socio-economic populations through trusted sources. The association between knowledge and practices demonstrates the importance of accurate public health communication to optimally follow preventive measures, such as structural interventions to address poverty and employment policies to address the unemployment crisis are required. Surveillance activity is needed to understand the actual behavior change among the population.

## Introduction

The coronavirus disease 2019 (COVID-19) has had a devastating effect globally since it was first identified in China in December 2019 (1). The acceleration of the transmission is confirmed by the fact that while it took 1 month since December 31, 2019 for the number to reach 10,000, and by March 6, 2020, over 100,000 cases were reported (2, 3). Toward the end of March 2020, there were 528,025 cases and 23, 669 deaths due to COVID-19 reported in over 190 countries (4).

Governments across the globe applied a series of behavioral interventions in the countries to minimize the transmission and burden of COVID-19 on the healthcare system and contain the transmission. These included infection prevention and control measures, that is, promotion of the use of masks along with following regular and thorough hand hygiene practices through handwashing with soap and water or alcohol-based hand rub, international and internal travel-related restrictions, and following social distancing (5, 6). Although randomized controlled trials (RCTs) demonstrate that personal protective measures such as hand hygiene and face masks have a small effect on respiratory infection transmission, higher compliance in a severe pandemic might improve the effectiveness (5, 7–9). However, adoption of such protective behaviors for curbing the spread of influenza and social distancing policies were reported of being of uncertain effectiveness, expensive, unpopular, difficult to implement (10–12), and highly disruptive to society (5, 13). However, in the absence of a vaccine, behavioral strategies for reducing the transmission of COVID-19 are vital to the global pandemic response (14). The efficacy and impact of these strategies depend on the compliance of the community and their cooperation. Historically, the adoption of such behaviors has depended on many factors related to personal perceptions and beliefs about the effectiveness of the preventive measures, the perceived risk of contracting the disease by self or family, and the perceived severity of health and economic consequences (15–18).

According to the Government of India (GoI) and the World Bank, 22% of population in India is poor which means 1 in 5 Indians is poor, with 80% of the poor population residing in rural areas (19, 20). COVID-19 has directed renewed attention to the informal employment sector of India, the migrant poor who move, often seasonally, from the villages to cities in search of work, and who in troubled times like these seek to return to villages where they feel more secure and have greater access to food and shelter (21–23). However, in rural and semi-urban communities, owing to family structures, close-knit communities, adherence to social isolation, preventing social gatherings, following social distancing behaviors may pose practical, motivational, and social barriers.

Therefore, this study aimed to explore determinants affecting adherence to protective measures against COVID-19 infection among communities in rural settings in India. A better understanding of behaviors, beliefs, concerns, knowledge, and associated predictive factors of people, during an emerging pandemic, is of crucial importance for public health officials and decision-makers, to enhance communication efforts for the promotion of individual and community health.

## Methods

### Study Design

A telephonic cross-sectional survey was conducted between June 5 and July 16, 2020.

### Study Setting and Participants

Satara and Sangli districts in the western region of Maharashtra were selected for the study purposively to have access to the rural population. Satara district is divided into 11 subdivisions and has a population of 3,003,741, whereas Sangli district is divided into 10 subdivisions and has a population of 2,822,143 persons. The rural population is 74.51 and 81.01% for Sangli and Satara districts, respectively (24, 25). The four purposively selected villages (clusters) in the Karad block were Khubi, Gondi, Shere, and Dushere which are rural, and the Karad Panchayat is considered semi-urban. The fifth village Lavanmachi was selected from the Walwa block of Sangli district.

### Sample Size

To assess the level of awareness about COVID-19, using a confidence level of 95%, the margin of error of 3.5%, and 50% awareness for COVID prevention measures, the sample size was estimated to be 800, adding 30% non-responsive to reach at a sample size of 1,000. Further, the sample size was adjusted by 30% to account for the households that had wrong contact numbers or for the contact numbers that were not reachable on the phone. The final sample size was 1,300.

### Sampling

Community support was sought from the village heads, local health officials, officials at the municipal corporation office, and police department before the study was initiated to gain access to household data and contact people on the telephone. The local police were informed of the commencement of the telephonic survey to keep them in the loop in case any of the respondents filed complaints about receiving a request for a telephonic survey. The local health authorities were informed not for any kind of penalty to the participant but only for keeping authorities in the loop for conducting a telephonic survey in the community.

A line list of all households in the six clusters was procured with support from the village head and the local government offices. The list included the name of one adult person in the household, their address, and contact number. A list of 1,300 households to be contacted was selected from these six clusters using a random number list. After contacting the household, they were asked to provide information on the number of adult members in the household, further, the Kish grid method (26) was used to select one adult respondent from each of these households randomly, and then, they were interviewed on the contact number provided in the household list or if they preferred to be called on a different number, this was noted and they were contacted on a number that they provided. Individuals, 18 years of age and above, currently residing in the study clusters, and who could understand and respond in Marathi, were eligible for participation.

### Survey Instruments

A semi-structured questionnaire was used for data collection. It explored demography (age, sex, education, source of income, and family size), knowledge and level of awareness of the community about COVID-19 infection, such as transmission routes, symptoms, prevention, and treatment measures, practices related to handwashing, wearing a face mask, using hand sanitizers, following social distancing, reducing physical contact, perceived risk of acquiring infection, the susceptibility of acquiring infection when at crowded places, sources of information about COVID-19 infection, and trust of participants in these information systems and sources.

### Data Collection

**Figure 1:**
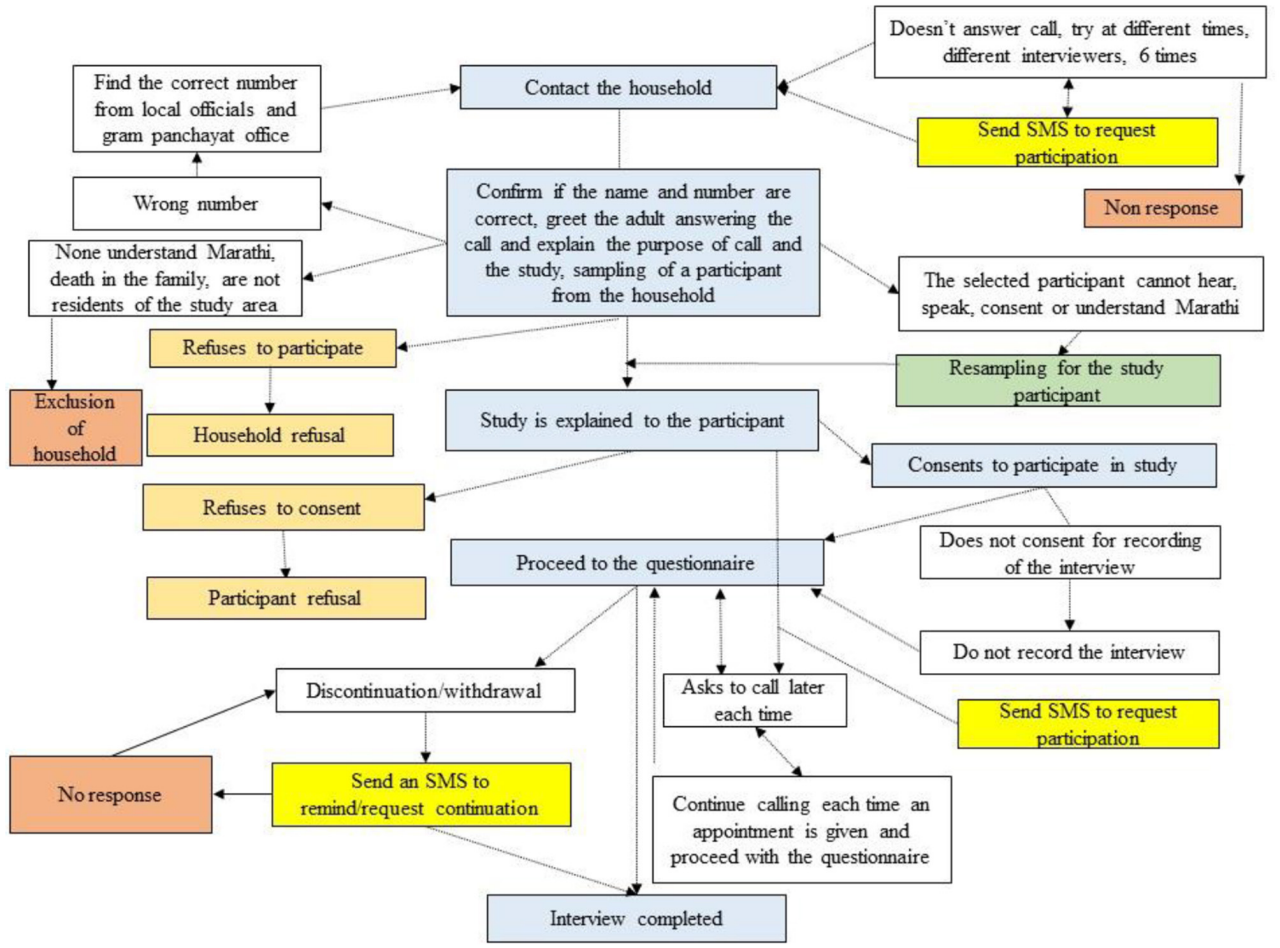
The steps involved in contacting a household, selecting a participant, and completing the interview.

In the survey, 12 interviewers were trained over 4 weeks for data collection. Pilot testing of the telephonic survey was completed between May 15 and May 31, 2020 by the trained 12 interviewers. Each household was approached using the contact number from the household list. Adults answering the call were given information about the study. One participant from each contacted household was selected randomly using the Kish method. In case the participant selected did not understand or respond in Marathi or not able to provide the informed consent (not able to hear or speak), resampling of the participant from the same household was conducted. At least six attempts were made to contact the household or the participant at different times (Table 1). The participants were interviewed as per their availability between 8:00 a.m. and 10:00 p.m. and the survey lasted for approximately 30 minutes. If the adult in the household was selected or if the participant refused to participate, they were considered as household or participant refusal, respectively. If participants decided to withdraw their participation from the study after consenting then they were counted as “discontinued.” In case, the male members or the head of household refused to give an appointment for the female members to be interviewed from the family, they were offered an option for a female surveyor to call them at a time convenient to them. An SMS was sent out to a household contact or participants to request their participation in the study, to participants who never answered calls or continued asking the surveyor to call them back later each time they called, and to those who disconnected calls or refused to participate before hearing about the study and ignored subsequent calls from the study team. Figure 1 illustrates steps involved in contacting a household and selecting a participant, and completing the interview.

**Table 1:** No. of phone call attempts made and the interview status.

**Interview status**	**No. of phone call attempts made**					
	**1**	**2**	**3**	**4**	**4 and above**	**Median (Range)**
Complete (956)	134	190	165	96	371	3 (1–54)
Discontinued (60)	4	3	5	3	45	9 (1–32)
Refusal by participants (73)	7	16	8	10	32	4 (1–24)
Household refusal (147)	12	21	16	14	84	5 (1–32)
Never reachable (99)					99	10 (1–50)
Wrong number (132)	13	14	9	11	85	6 (1–20)
Excluded (12)	5	0	0	1	6	3 (1–13)

### Ethics Approval

The study was approved by the Institutional Ethics Committee of ICMR-National AIDS Research Institute, Pune, Krishna Institute of Medical Science Deemed-to-be University, Karad, and KEM Hospital Research Center, Pune. The data were collected after verbal informed consent by the participant, and the survey was audio-recorded if the participant consented.

### Measurement of Variables

1) Demographic information, such as questions about age in completed years, sex, current place of residence, level of education completed, and the main source of income in the last 12 months.
2) Knowledge and level of awareness of the community about COVID-19 infection, such as questions on transmission routes, symptoms, availability of prevention, and treatment measures, perception on complete recovery, duration of transmission of infection to others, knowledge about the high-risk populations, measures to prevent COVID-19 infection, and if they had heard about social distancing.
3) Practices related to the protective measures adopted for COVID-19, such as using soap for handwashing and sanitizer while being outdoors, wearing masks, following social distancing, and reducing physical contact (staying indoors).
4) Perceived risk of acquiring infection and susceptibility.

### Data Analysis

#### Independent Variables

A standard descriptive summary for age, family size was expressed in percentages or as the mean and SD. Education was categorized as—never attended school, primary, upper primary, secondary, senior secondary, undergraduate, post-graduate and above, and vocational training. The variable of self-risk perception was categorized as—yes, no, and do not know. The frequency for looking for COVID-19 updates on media was categorized as—once a day, many times a day, and not every day/never. Feeling worried about having COVID-19 symptoms as—worried/very worried, somewhat worried, and rarely/not worried. Knowledge scores ranged from 0 to 24; this was included as a continuous variable based on 13 questions related to knowledge of prevention measures for COVID-19.

#### Dependent or Response Variable

The responses indicating proper use of protective measures were coded as 1 and otherwise 0. This included an “**Always**” response to questions on wearing a mask while going outside the house, washing hands with soap and water when coming from outside, using hand sanitizer while outside or after coming home, while a “NO” response to attending social gatherings in past 15 days. Combining the responses to these variables, a variable was generated to capture the data about failing to use/follow any of these protective measures. Correlates of failing to use protective measures were identified using univariate and multivariate logistic regression models. A multivariable logistic regression analysis was performed to identify factors related to knowledge, attitudes, and practice. A multivariable regression model was used to understand socio-demographic and attitudinal correlates of not following protective measures related to COVID-19. All analyses were done using STATA software; version 16.0 (Stata Corp. 2019. Stata Statistical Software: Release 16. College Station, TX, USA: Stata Corp LLC.).

## Results

A total of 1,479 households/individuals were contacted for the study, of which 220 households and participants refused to participate in the study, 132 contact numbers were incorrect, 100 potential participants were never reachable, and 11 households were excluded from the study. The final sample consists of 1,016 respondents. Of these, 956 participants had completed all the items in the interview and 60 participants completed the interview partially. The response rate was 69%. In order to complete the survey, 8,532 calls were made. Table 1 describes the number of phone call attempts made and the outcome of the interviews.

Of the enrolled participants 72% (734) were men. The higher representation of male participants in the study may be a coincidence. Nearly half of the participants were in the age group between 30 and 50 years, 39% of the participants had completed secondary education, 28% were self-employed or owned a business followed by 24% salaried, and 14% of the participants reported farming to be the main source of income (Table 2). The missing information is not presented in the data.

**Table 2:** Socio demographic characteristics of the study participants.

**Characteristics**	***N* = 1016** **No. of participant (%)**	**Characteristics**	***N* = 1016** **No. of participant (%)**
**Gender**		**Age (years)**	
Male	734 (72)	Mean (SD)	46.2(13.8)
Female	282 (28)	<30	148 (15)
**Education**		31 to 40	241 (24)
Never attended school	53 (5)	41 to 50	254 (25)
Primary	71 (7)	51 to 60	200 (20)
Upper primary	104 (10)	>60	169 (17)
Secondary	251 (25)	**Occupation**	
Senior secondary	147 (14)	Business/ Self employed	282 (28)
Undergraduate	276 (27)	Salaried (Private/Govt.)	252 (25)
Post graduate and above	73 (7)	Agriculture	144 (14)
Vocational training	36 (4)	Laborer^*^	104 (10)
**Family size**		Retired	97 (10)
One or Two	82 (8)	Unemployed	58 (6)
3 to 6	692 (68)	Student	47 (5)
More than 6	174 (17)	Refused/ missing	19 (2)
Median (IQR)	4 (4, 6)	Home Maker	13 (1)

* *Laborer included agriculture labor, casual, maid and other. The total number of participant will differ in each category due to non-response*.

### Knowledge of Transmission of COVID-19 Infection

The findings showed that 94% (955) of the respondents had correct knowledge of COVID-19 transmission, and 97% (988) knew that it could be transmitted through coughing, sneezing, and close physical contact. The knowledge regarding the symptoms of COVID-19 showed that 65% (664) of the population knew that the dry cough, fever, and shortness of breath (all) could be symptoms of COVID-19. The major source of COVID-19 related information was reported to be television (91%), local announcements (84%), and local healthcare providers (82%); however, a great deal of trust was more of local healthcare providers (65%) and local announcements 49%. Tables 3A–5 show responses to items related to knowledge, attitude, and practices toward COVID-19.

**Table 3A:** Participants knowledge and their level of awareness related to COVID- 19 infection.

**Response category** **(correct response)**	**No. of correct** **responses (%)**
**Transmission of coronavirus infection**	
Through coughing, sneezing, close contact (Yes)	988 (97)
Transmissible from person to person (Yes)	955 (94)
Mosquito bite (No)	921 (91)
Through food or water (No)	780 (77)
Not transmissible (No)	663 (65)
Transmitted by animals to human only (No)	602 (59)
**Symptoms of coronavirus infections correctly mentioned**	
Fever	898 (88)
Shortness of breath	803 (79)
Dry cough	798 (79)
All (dry cough, fever, shortness of breath)	664 (65)
**Knowledge about measures to prevent coronavirus infection**	
Maintain personal hygiene and frequent hand washing with soap and water	983 (97)
Obey the advisories issued by government and health administrations	980 (96)
Wearing mask when sick or having some symptoms	969 (95)
Maintain social distancing	968 (95)
Avoid traveling to known affected area	939 (92)
Avoid touching your eyes, nose and mouth with unwashed hands	911 (90)
All the above	832 (82)

**Table 3B:** Participants reporting sources of information for Covid and level of trust.

**Source of information**	**Individuals reporting source** ***n* (%)**	**Very little trust** ***n* (%)**	**Great deal of trust** ***n* (%)**
Television	929 (91)	52 (6)	378 (41)
Local announcement	850 (84)	52 (6)	406 (48)
Local healthcare providers	837 (82)	22 (3)	542 (65)
WhatsApp/Facebook/Twitter	723 (71)	165 (23)	102 (14)
Newspaper	691 (68)	38 (6)	184 (27)
Local groups	602 (59)	46 (8)	239 (40)
Web based information	508 (50)	53 (10)	143 (28)
Other (family, friends, relatives, known contacts)	173 (17)	–	–
Other (Social workers in village)	36 (3.5)	–	–

**Table 4:** Responses of participants to attitudinal statements regarding COVID 19.

**Statement**	**No. of responses (%)**
A person with coronavirus infection disease recover completely	890 (88)
Coronavirus infection is completely preventable at present	783 (77)
Availability of specific treatment at present	308 (30)
**Risk perception**	
**High-risk population for coronavirus infection**	
Elderly people (above 60 years of age)	956 (94)
Contact with confirmed COVID positive case	945 (93)
Recent travel history to the affected area	918 (90)
Persons with preexisting morbidity	891 (88)
Pregnant women and children	860 (85)
All of the above	725 (71)
**How susceptible do you consider yourself to an infection**	
Very highly/ somewhat susceptible	390 (38)
Not at all susceptible	470 (46)
**Chances of getting infected in crowded places**	
Very high chance	479 (47)
Somewhat high chance	337 (33)
Very less chance	101 (10)
No chance	78 (8)
**Social distancing can break the spread of coronavirus infection**	
Yes, definitely	569 (56)
Yes, somewhat	301 (30)
Yes, but very little chance	60 (6)
No, not at all	36 (4)
Do not know	29 (3)
**How long a person infected can spread coronavirus**	
< = 4 days	49 (5)
< = 8 days	72 (7)
10 to 12 days	45 (4)
13 to 15 days	499 (49)
16 to 21 days	25 (2)
**Chances of getting infected in crowded places**	
Very high chance	479 (47)
Somewhat high chance	337 (33)
Very less chance	101 (10)
No chance	78 (8)
**Heard about social distancing**	
Yes	863 (85)
**Opinions about the meaning of the word “social distancing”**	
Avoiding rush at workplaces	934 (92)
Avoiding shaking hands	915 (90)
Keeping 2 meter distance from people	915 (90)
Avoiding social gatherings	894 (88)
Avoiding public places	866 (85)
Avoiding going out of the house	776 (76)
All of the above	643 (63)

**Table 5:** Reported practices and behavior related to COVID 19.

**Practice**	**No. of responses (%)**
Wash hands with soap and water after coming home from outside	967 (95)
Wearing a mask ALWAYS while going outside the house	953 (94)
Feels necessary to ALWAYS cover your face/mouth while coughing or sneezing	923 (91)
Always using hand sanitizer while outside	779 (77)
In past 15 days, attended social gatherings visited friends for tea, socializing	95 (9)
**Looking for updates on social media**	
Once in a day	223 (22)
Many times a day	650 (64)
Not every day/never	67 (7)
**Feel worried by COVID symptoms**	
Worried/very worried	375 (37)
Somewhat worried	240 (24)
Rarely/not worried	328 (32)

### Attitudes of Respondents Toward COVID-19

The attitudes of the respondents toward COVID-19 were assessed, and the results (Table 4) showed that 88% (890) of the respondents believed that persons having COVID-19 infection can recover completely and, 77% (738) had felt that COVID-19 infection is completely preventable at present; however, 30% believed that there is the availability of specific treatment of COVID-19 at present. Most respondents reported that the persons who have traveled to an area affected by COVID-19 (90%) have come in contact with a person having the infection (93%), and elderly persons above 60 years of age (94%) are the “high risk” population for COVID-19 infection. In addition, 46% (470) did not consider themselves as susceptible to the infection and approximately half 49% (499) felt that a person having COVID-19 infection would transmit the infection to others up to 13–15 days. Nearly, one-fifth of the participants (21%) did not know for how long a person who has an infection could transmit it to others. Additionally, only 56% (569) of the respondents felt that following social distancing can break the spread of COVID-19 infection. Approximately 81% of respondents believed that the lockdown was an important strategy to prevent the spread of COVID-19.

### Practices Related to Social Distancing

The survey results showed that a total of 95 participants (9.4%) have attended a social gathering and visited a friend for tea/discussion in the last 15 days. Of the total 95% (967) reported immediate washing of hands after returning home, followed by 94% (953) stating wearing a mask while going outside, and always using a hand sanitizer while being outside was reported by 77% (779) of the participants.

### Stress

The current situation was stressful for families, and feeling lonely due to the pandemic situation was reported by 55 and 49% of the respondents, respectively. Additionally, 24% (241) reported feeling angry and more anxious than in the past. Furthermore, 16% reported having faced difficulty in availing healthcare due to lockdown.

### Association Between Socio-Demographic Variables and Not Following Protective Measures

Association between socio-demographic variables and not following protective measures is described in Table 6. The socio-demographic variables, such as age, sex, and education, were not independently associated with risk-taking behavior (not following protective measures). Association between socio-demographic variables and not following protective measures showed no difference in peri-urban and rural settings. Not having any source of income was independently associated with not following protective measures for COVID-19 prevention AOR 1.5 (95% *CI* 1.01–2.3). Among men, “having no source of income” was associated with not following protective measures as compared with men who had a source of income [*OR* 1.8, 95% *CI*: 1.1–2.9, *p* = 0.015]. The knowledge score was independently associated with the risk of not using protective measures for COVID-19 prevention. With each unit increase in knowledge score, the risk of not using protective measures reduced by 16%.

**Table 6:** Association between socio-demographic variables and not following protective measures.

**Characteristic**	**No. of individuals** **(% out of 1016)**	**No. of individuals not following protective measures (%)**	**OR (95% CI)**	**AOR (95% CI)**	***P* value**
**Age group**					
30 & Below	148 (15)	42/143 (29)	1	1	
31–40	241 (24)	53/240 (22)	0.7 (0.4–1.1)	0.92 (0.5–1.6)	0.756
41–50	254 (25)	72/247 (29)	0.99 (0.63–1.6)	1.02 (0.6–1.7)	0.927
51–60	200 (20)	70/195 (36)	1.3 (0.85–2.1)	1.7 (0.99–2.9)	0.055
Above 60	169 (17)	64/162 (40)	1.6 (0.97–2.5)	1.4 (0.8–2.6)	0.221
**Gender**					
Male	734 (72)	222/715 (31)	1	1	
Female	282 (28)	79/273 (29)	0.9 (0.7–1.2)	1.01 (0.7–1.5)	0.948
**Education**					
Illiterate	53 (5)	16/49 (33)		1	
Primary	175 (17)	47/171 (27)	0.78 (0.4–1.6)	1.3 (0.5–3.2)	0.610
Secondary and senior secondary	398 (39)	121/388 (31)	0.93 (0.5–1.8)	1.9 (0.8–4.6)	0.142
Above senior secondary	385 (38)	117/379 (31)	0.92 (0.5–1.7)	2.3 (0.9–5.5)	0.075
**Having source of income**					
Yes	782 (77)	217/763 (28)	1	1	
No	215 (21)	79/211 (37)	1.5 (1.1–2.1)	1.5 (1.01–2.3)	0.048
**Family size (No. of members)**					
Small (1 to 4)	501 (49)	145/501 (29)	1	1	
Large (more than 4)	447 (44)	140/447 (31)	1.1 (0.8–1.5)	1.3 (0.9–1.7)	0.157
**Self-risk perception (consider susceptible to infection)**					
No	470 (46)	145/461 (31)	1	1	
Yes	390 (38)	116/389 (30)	0.93 (0.7–1.2)	1.1 (0.8–1.6)	0.496
Do not know	136 (13)	37/132 (28)	0.85 (0.6–1.3)	0.86 (0.5–1.4)	0.545
**Look for COVID update on media**					
Once in a day	223 (22)	65/223 (29)	1	1	
Many times a day	650 (64)	192/650 (30)	1.02 (0.7–1.4)	1.03 (0.7–1.5)	0.879
Not every day/never	67 (7)	24/67 (36)	1.4 (0.8–2.4)	1.03 (0.6–1.9)	0.924
**Feel worried by COVID 19 symptoms**					
Worried/Very worried	375 (37)	105/375 (28)	1	1	
Somewhat worried	240 (24)	61/240 (25)	0.87 (0.6–1.3)	0.7 (0.5–1.1)	0.082
Rarely/Not worried	328 (32)	117/328 (36)	1.4 (1.03–1.96)	1.2 (0.8–1.7)	0.410
**Knowledge**					
COVID 19 knowledge score(ranging from 1 to 24)	Mean (SD) 20.3 (2.4) Median(IQR) 21 (19, 22)	Mean (SD)19.7 (3.1)Median(IQR) 20 (18, 22)	0.86 (0.81–0.91)	0.84 (0.78–0.90)	<0.01
**Outbreak is stressful**					
No	390 (38)	115/390 (29)	1	1	
Yes	556 (55)	167/556 (30)	1.03 (0.77–1.36)	1.01 (0.74–1.4)	0.931
**Locality**					
Peri-urban	864 (85)	260/841 (31)	1	1	
Rural	150 (15)	40/145 (28)	0.85 (0.57–1.26)	0.9 (0.6–1.4)	0.641

## Discussion

This study was conducted to explore the determinants affecting compliance to protective measures against COVID-19 infection among rural and semi-urban communities in the western region of Maharashtra, India. The study highlighted high knowledge about COVID-19 among rural and semi-urban communities. The findings in this survey suggest socio-demographic factors that influence the adherence to the protective measures for COVID-19 prevention and government advisories that would prove useful in planning behavioral change communication programs for containment of the current COVID-19 pandemic and also new emerging infectious diseases in these regions.

The seroprevalence of COVID-19 showed an increase between May and August 2020 in India (27). The third round of the serosurvey conducted in India in August–September 2020 and December 2020–January 2021 showed an increase in seroprevalence in the urban areas, while the rural population is still at risk and surveillance has been recommended (27, 28). We conducted an epidemiological survey aimed at assessing knowledge, attitudes, and practices and identifying opportunities to target interventions to contain the spread of COVID-19 infection in rural and semi-urban regions of India. When compared with a study conducted in Nigeria (29), most of the study participants reported accurate knowledge and compliance with following the protective measures. The majority of the current study participants reported maintaining social distancing, frequent handwashing with soap and water, wearing a mask while leaving the house, and obeying government advisories. The study conducted by Dkhar et al. in April, 2020 among social media users in Jammu and Kashmir, showed similar results that respondents exhibited good knowledge, positive attitudes regarding COVID-19 during the pandemic with most of them reporting regularly wearing masks, washing hands with soap and water regularly, following lockdown guidelines, and maintaining social distancing (30). Similarly, cross-sectional online survey conducted in India also showed the correct rate of knowledge (74.7%), perception (57.6%), and practices (88.1%) toward COVID-19 (31). While closer to the outbreak, reports showed poor attitudes toward disease prevention and control in Thailand (32).

The current study was conducted between the fourth and fifth phase of lockdown in the month of May and June 2020 with unlock being initiated in the state of Maharashtra at this time. The accurate knowledge of COVID-19 reported by the participants and compliance with following personal protective measures in this study could be attributed to the months-long campaigning efforts targeted toward making messaging more effective through pre-recorded public local announcements and using locally available resources, such as rickshaw/tempo in the rural areas of Maharashtra, India (33). In addition, local news resources have reported that community social workers are utilizing innovative and simplified ways of using umbrellas to explain social distancing (34). Several regions during this data collection period were considered as containment and micro-containment zones where the village borders and also smaller localities were sealed. The local news resources reported that this led to fear and panic among the communities (35, 36). This may imply active observation and discussions within groups in these study areas, facilitated through the local healthcare providers, social workers, and local announcements, which have the trust of the community. GoI launched a “*jan andolan*” (public campaign) for COVID-19 appropriate behaviors (37). The participants in the current study scored 90% (median score) for the efforts of the state government to contain the pandemic.

Although participants reported having good knowledge about preventive measures, 46% perceived no risk of acquiring COVID-19 infection. Low self-risk perception is contrary to the findings reported during the early stage of the pandemic in China, where studies showed more than 70% of the respondents were worried about them or a family member acquiring the infection (38–40). Similar to the current study findings, low-risk perception (median score of 5 out of 10) was reported in a study conducted in the United States (41). This low self-risk perception in the present study is an indication of complacency that might set in once prevention fatigue rises in the community. It could also result in vaccine hesitancy. Therefore, local communication strategies should emphasize creating public awareness and bringing about a behavior change through population tailored interventions to help communities sustain following protective measures, since, it is likely that adherence to protective measures may not be sustained when the penalties are revoked. Further, novel approaches to estimate compliance with lockdown measures in the COVID-19 pandemic may be adopted (42). In addition, face masks are proposed to be the most obvious measure to prevent transmission and they can generate peer pressure kind of response in the communities (43). It would be important to continue with efforts for personal protective measures to avoid a false sense of security among those who receive vaccine which is currently being rolled out (43).

This study highlighted the evidence about the source of COVID-19 related information for the community and their level of trust in them. For 91% of the participants, television was the source of COVID-19 related information, local announcements 84%, local healthcare providers 82%, and social media 71%; however, participants had a great deal of trust in the healthcare providers and local announcement systems. Similarly, Zhong et al. also reported that social media was a primary source for COVID-19 information, whereas the most trusted sources were healthcare professionals (40). Therefore, these sources must be involved while delivering health information and interventions tailored to the needs of the community.

Mental health concerns and treatment are left out when the limited resources are mobilized for pandemic containment (44). History suggests that any infectious disease outbreak or pandemic brings with it a major setback in the mental health front. In 2014, during the Ebola outbreak, anxiety-depression and symptoms of post-traumatic stress disorder (PTSD) were more prevalent even after 1 year of Ebola response (45). Mental health concerns, such as stress, anxiety, depression, insomnia, denial, anger, and fear were reported by Roy et al. through a scientific review (44). In the context of India, mental health concerns of the COVID-19 pandemic may be more complex due to a large proportion of the socially and economically vulnerable population, migrant workers, and laborers who have been reported to be affected the most. In India, within hours of the lockdown announcement on March 25, 2020, millions of migrant laborers began reverse migration (46, 47). The phenomenon produced loneliness, panic, fear, feelings of isolation, and economic anxiety. The migrant workers having a serious nervous breakdown and depressive psychotic disorders were reported in the media (48). In the current study, more than half of the participants (55%) reported that this period was stressful for the family as they experienced loneliness and suffered “more stress and anxiety” than in the past.

In the current study, it was reported that persons with no source of income were not following the protective measures. The spike of COVID-19 infections in rural areas in Maharashtra was attributed to the reverse migration of workers returning from the urban areas (49) and until September 2020, rural areas contributed to 49.7% of all cases in the country, and Maharashtra being the major destination state for reverse migration for migrant laborers (50, 51). In Australia, during a pandemic influenza outbreak, it was reported that individuals who are employed but not able to work from home are less likely to report intended compliance with quarantine restrictions (52). On the contrary to the current study findings for a swine flu outbreak in the United Kingdom in 2009, where participants who were not employed, were poor, had an annual household income of less than GBP £30,000, or had no educational qualifications were significantly more likely to adopt avoidant behaviors (e.g., avoiding large crowds or public transport) (53).

In the present study, 28% of the participants were self-employed, 14% were engaged in agricultural activities as their main source of income, and 10% worked as laborers. The government restricted commercial and industrial activity and imposed a ban on the movement of people and goods deemed “non-essential” from March 25, 2020 that affected the income-generating activities. During the months of April and May, 2020, these exemptions were maintained and further supported by opening up agricultural input stores, machinery repair shops, and agribusinesses. Inter-district travel was prohibited other than for emergency purposes, and public transport facilities remained shut down until mid-May, 2020, and the movement of people, such as agricultural laborers, remained severely constrained (54). This necessitates the need for attention to the underserved and marginalized populations, and people from low socio-economic status to prevent long-lasting adverse health outcomes.

This study was conducted at a time when there was a complete lockdown and no one was venturing out. We had success in conducting large-scale telephonic surveys in rural and peri-urban settings. The data collection for this study was conducted using telephone calls, therefore, the households that did not have a telephone were not included in the study. Furthermore, homeless populations might not have been enumerated in the gram panchayat and Nagar panchayat list and therefore may have been missed from the study. Another limitation with the telephonic method of data collection would have been that participants may be reluctant to speak with an unknown caller, leading to household and participant refusals. It is natural to have a shorter attention span over telephonic interviews than in face-to-face interviews. Therefore, there were few missing data and discontinued interviews in this study. Since this was a telephonic survey, we had to rely on self-reported instead of observed practices, thus were unable to verify whether the responses were affected by social desirability bias.

A positivist approach was used and, therefore, we included all socio-demographic and behavioral factors that could influence corona appropriate behaviors in the community. However, cultural and religious factors were not explored which was a limitation. These factors would have been difficult to explore on the telephone. Qualitative exploration was not possible considering the situation. Therefore, these factors were not explored in order to prevent any adverse comprehension by the interviewee. A face-to-face in-person interviewing was not possible due to the travel restrictions and social distancing guidelines. Owing to the lockdowns and inaccessibility to the study participants except through telephone, the Kish method was the most feasible method of data collection. However, the anticipated high intra-cluster similarity may have weakened the generalizability of the results.

## Conclusion

The study shows that the lower knowledge score and having no source of income were independently associated with the risk of not following COVID-19 preventive behaviors. The COVID-19 public health interventions and behavior change communication strategies should be specifically directed towards the low socio-economic populations through the trusted sources, such as structural interventions to address the poverty and employment policies to address the unemployment crisis. The association between knowledge and practices demonstrates the importance of prompt and accurate public health communication to follow preventive measures optimally.

Although protective measures during the study duration were high, surveillance activity is needed to understand the actual behavior change among populations. Local interventions to mitigate the effect of mental health concerns in this population are necessary. Perception of risk should be encouraged, and risk communication should be tailored to this rural population considering mental health while developing these strategies.

## Data Availability Statement

The original contributions presented in the study are included in the article/supplementary material, further inquiries can be directed to the corresponding author/s.

## Ethics Statement

The study was approved by the Institutional Ethics Committees of ICMR-National AIDS Research Institute, Pune, Krishna Institute of Medical Science Deemed-to-be University, Karad, and KEM Hospital Research Centre, Pune. The data was collected after verbal informed consent by the participant, the survey was audio-recorded if the participant consented.

## Author Contributions

SS, SJ, and SR were involved in the conceptualization of the study. The study methodology was developed by SS, SJ, SR, RK, SB, and SPS. SPS, SR, RK, and SS trained the team for data collection. SPS, RP, and AJ implemented the study. The data were curated by SPS and RP. The data analysis plan was developed and the data were analyzed by SSS, SPS, DU, and SS. SPS and SS wrote the first draft of the article. All authors contributed to reviewing, editing the drafts, and approving the manuscript.

## Conflict of Interest

The authors declare that the research was conducted in the absence of any commercial or financial relationships that could be construed as a potential conflict of interest.

## Publisher's Note

All claims expressed in this article are solely those of the authors and do not necessarily represent those of their affiliated organizations, or those of the publisher, the editors and the reviewers. Any product that may be evaluated in this article, or claim that may be made by its manufacturer, is not guaranteed or endorsed by the publisher.
